# Atrial Strain Assessment for the Early Detection of Cancer Therapy-Related Cardiac Dysfunction in Breast Cancer Women (The STRANO STUDY: Atrial Strain in Cardio-Oncology)

**DOI:** 10.3390/jcm12227127

**Published:** 2023-11-16

**Authors:** Daniela Di Lisi, Antonella Moreo, Grazia Casavecchia, Christian Cadeddu Dessalvi, Corinna Bergamini, Concetta Zito, Cristina Madaudo, Rosalinda Madonna, Matteo Cameli, Giuseppina Novo

**Affiliations:** 1 Department of Health Promotion, Mother and Child Care, Internal Medicine and Medical Specialties (ProMISE), University of Palermo, Piazza delle Cliniche, 2, 90127 Palermo, Italy; 2 Division of Cardiology, University Hospital “Paolo Giaccone”, Via del Vespro 129, 90127 Palermo, Italy; 3 Cardiology IV, “A. De Gasperis” Department, ASST Grande Ospedale Metropolitano Niguarda, 20162 Milan, Italy; 4 Cardiology Unit, Department of Medical and Surgical Sciences, University Hospital of Foggia, University of Foggia, 71122 Foggia, Italy; 5 Department of Medical Sciences and Public Health, University of Cagliari, 09042 Cagliari, Italy; 6 Department of Medicine, Division of Cardiology, University of Verona, 37129 Verona, Italy; 7 Department of Clinical and Experimental Medicine, University of Messina, 98122 Messina, Italy; 8 Division of Cardiology, University Hospital “Gaetano Martino”, 98124 Messina, Italy; 9 Department of Pathology, Cardiology Division, University of Pisa, 56124 Pisa, Italy; 10 Department of Medical Biotechnologies, Division of Cardiology, University of Siena, 53100 Siena, Italy

**Keywords:** cardiotoxicity, cardio-oncology, GLS, left atrial strain, chemotherapy, CTRCD

## Abstract

Left ventricular global longitudinal strain (GLS) has an important role in the diagnosis of cancer therapy-related cardiac dysfunction (CTRCD). Little is known about the role of atrial function in diagnosing CTRCD. The aim of our study was to assess the impact of anti-cancer drugs on atrial function measured by speckle-tracking echocardiography in breast cancer women. A prospective multicenter study was conducted enrolling 169 breast cancer women treated with anthracyclines. A cardiological evaluation including an electrocardiogram and echocardiogram with an analysis of GLS, left atrial (LA) strain, and LA stiffness (LASi) was performed at baseline (T0), 3 (T1), and 6 months (T2) after starting chemotherapy. The patients were divided into two groups: patients with asymptomatic mild cardiotoxicity at T1 (with a relative reduction in GLS > 15%; Group 1) and those without (Group 2). We did not find a significant change in left ventricular ejection fraction (LVEF) at T1 and T2; we found a significant change in GLS (*p*-value < 0.0001) in the peak atrial longitudinal strain (PALS) and in LASi (*p*-value < 0.0001). Impairment of atrial function was greater in Group 1 compared to Group 2. A PALS variation > 20.8% identified patients who were most likely to develop asymptomatic mild cardiotoxicity [AUC 0.62; CI (0.51–0.73) *p* = 0.06, sensitivity 45%, specificity 69.5%]. Conclusions: PALS and LASi significantly change during chemotherapy in association with GLS. Atrial strain is an additional parameter that could be measured together with GLS to detect cardiotoxicity early.

## 1. Introduction

It is known that anti-cancer drugs can cause cardiovascular complications such as left ventricular dysfunction, heart failure, arrhythmias, myocardial ischemia, and arterial hypertension. The mechanisms underlying cardiotoxicity may involve direct and/or indirect mechanisms of cardiomyocytes. Anthracyclines are associated with a high incidence of heart failure as a consequence of direct damage to cardiomyocytes which, once their function is altered, could lead to cell death. Other mechanisms underlying cardiotoxicity may include vascular dysfunction and thromboembolic ischemia. Other adverse effects result from impaired signaling of cardioprotective factors for cardiomyocytes, such as Neuregulin-1 (NRG1), or for other cardiac cell populations, such as vascular endothelial growth factor (VEGF) and platelet-derived growth factor (PDGF) [1,2,3,4]. Several definitions of cardiotoxicity have been previously proposed [5,6]. The 2022 ESC guidelines on cardio-oncology have optimized the practical management of the cardio-oncology patient and have proposed a universal definition of cardiac dysfunction related to anti-tumor therapy (CTRCD), distinguishing it into symptomatic and asymptomatic [7,8]. Asymptomatic mild CTRCD is defined as the presence of preserved LVEF ≥ 50% but a new relative decline in left ventricular global longitudinal strain (GLS) > 15% from baseline and/or a new rise in cardiac biomarkers [7]. Several studies conducted in the field of cardio-oncology have shown that LVEF alters late during anti-cancer therapy [9], whereas GLS alters earlier by identifying subclinical cardiac damage [10]. Cardiac biomarkers (natriuretic peptides and high-sensitivity troponins) can also detect cardiotoxic damage at an early phase and possibly predict the future development of CTRCD [11,12]. A recent systematic review and meta-analysis showed that measuring GLS after initiating potentially cardiotoxic chemotherapy with anthracyclines with or without trastuzumab had good prognostic performance for subsequent CTRCD [13]. Thus, GLS should be assessed before starting chemotherapy and during chemotherapy to detect early cardiac damage and to start cardioprotective therapy [14]. While the role of GLS in cardio-oncology is well established, little is known about the role of left atrial (LA) strain. Few studies have analyzed changes in atrial function in cancer patients. LA strain is known to have clinical utility in a variety of settings. For example, LA reservoir strain was introduced as a standard parameter in the diagnostic algorithm of diastolic function in HFpEF [15]. Indeed, LA strain is known to be an early and sensitive marker of myocardial fibrosis and elevated left ventricular (LV) filling pressures [16]. LA strain also has a prognostic role in patients with heart failure. Mandoli et al. showed that the loss of left atrial contractile function predicts worse outcomes in heart failure patients with reduced LVEF (HFrEF) [17]. Even in patients with cardiac amyloidosis and HFpEF, a loss of atrial contraction and atrial electromechanical dissociation in patients with sinus rhythm (SR) was associated with a poorer prognosis than in patients with SR and effective mechanical contraction [18]. There is a close correlation between GLS and LA strain. LA reservoir and pump strain are mainly determined by GLS and filling pressure, as demonstrated by Inoue et al. [16]. Thus, considering the close correlation between GLS and LA strain and the changes in GLS during chemotherapy, the aim of our study was to assess the impact of anthracyclines and trastuzumab on atrial function measured by speckle-tracking echocardiography (STE) in breast cancer women. 

## 2. Materials and Methods

### 2.1. Centers Involved 

A prospective multicenter study was carried out enrolling 169 women with newly diagnosed breast cancer, scheduled to receive anthracyclines (ANT) therapy followed by paclitaxel plus trastuzumab. Eight different Italian centers were involved: University Hospital “Paolo Giaccone”—Palermo (64 patients), ASST Metropolitan Hospital “Niguarda”—Milan (28 patients), University Hospital “Ospedali Riuniti”—Foggia (26 patients), University Hospital “San Giovanni di Dio”—Cagliari (23 patients), University Hospital “Borgo Trento”—Verona (16 patients), University Hospital “Gaetano Martino”—Messina (12 patients), University Hospital of Pisa, and University Hospital of Siena. 

### 2.2. Criteria for Enrolling Patients in the Study 

Inclusion criteria were newly diagnosed breast cancer, age between 18 and 80 years old, and treatment with anthracyclines for at least 4–6 cycles followed by trastuzumab. Exclusion criteria were prior chemotherapy or radiotherapy; ischemic heart disease (prior myocardial infarction and/or coronary revascularization), severe valve diseases, baseline LVEF < 50%, atrial fibrillation and sustained ventricular arrhythmias, life expectancy < 1 year, poor acoustic window, and unfeasible STE. 

### 2.3. Follow-Up and Study Methodology 

After approval by the local ethics committee and after providing written informed consent, all the patients underwent cardiological evaluation at baseline (T0), 3 (T1), and 6 (T2) months after starting chemotherapy. Clinical and anamnestic data were collected. An electrocardiogram and an echocardiogram with STE analysis were performed at baseline and during follow-up. The diagnosis of CTRCD was made according to the 2022 ESC guidelines on cardio-oncology, based on the LVEF and/or GLS reduction from baseline. Patients were divided into 2 groups: patients with mild asymptomatic cardiotoxicity, defined as a relative reduction in GLS > 15% at T1 compared to baseline (Group 1), and those without (Group 2). 

### 2.4. Echocardiographic Examination 

Echocardiographic examination was performed according to EACVI/American Society of Echocardiography (ASE) recommendations for chamber quantification [19], using a General Electric (GE) Vivid E95 ultrasound system prime echocardiography machine and a 4Vc-D (1.4–5.2 MHz) linear transducer. LV systolic and diastolic function were assessed. LVEF was assessed using the biplane-modified Simpson method from the apical 4- and 2-chamber views. Diastolic function was assessed according to the EACVI recommendations on diastolic function [13,14,15,16,17,18,19]. LA volume was measured using the modified Simpson biplane method and was indexed to the body surface to obtain the LA volume index (LAVi). Early diastolic (E) and late diastolic (A) velocities were assessed by trans-mitral pulsed wave Doppler to calculate the E/A ratio; septal and lateral peak systolic (S’), early diastolic (E’), and late diastolic (A’) annular velocities were obtained by Tissue Doppler Imaging (TDI). The mean E/E’ ratio was calculated and used as an index of the LV filling pressure. The maximum velocity of tricuspid regurgitation was sampled to estimate systolic pulmonary pressure (sPAP) and assess the degree of diastolic dysfunction in addition to other parameters. Right ventricular function was assessed by tricuspid annular plane systolic excursion (TAPSE) and by TDI (peak systolic S’). 

#### Speckle-Tracking Echocardiography (STE)

STE analysis was performed offline to assess GLS and LA strain using semiautomated 2D Strain GE software (Echopac V.202, GE Healthcare, Horten, Norway, version for LA strain analysis: 2.02—release 34.0). The operators analyzing the images were not aware of the clinical characteristics of the patients. The GLS was obtained by recording apical 2-, 3-, and 4-chamber views with a frame rate between 60 and 80 fps and a stable electrocardiographic trace. The analysis was performed offline by two experienced and independent echocardiographers. Normal reference values of GLS were considered: −21.5 ± 2%, with a lower limit of normal of—18% [20,21]. For the evaluation of LA strain, STE analysis was performed on apical 2- and 4-chamber views optimized in terms of orientation, depth, and gain to avoid LA foreshortening and to visualize the entire LA throughout the cardiac cycle. The LA border was traced manually in these views, delineating a region of interest (ROI). The software divided the ROI into 6 segments and automatically calculated the LA reservoir strain (PALS—peak atrial longitudinal strain), peak atrial conduit strain, and peak atrial contraction strain (PACS). Indeed, the LA strain can be used to measure all LA functions during the cardiac cycle. QRS was used as the starting point to measure LA strain. LA strain was usually calculated as the average of all LA segments in 4- and 2-chamber views; only in a few patients with a non-optimal view of the left atrium in the 2-chamber view, it was calculated as the average of the segments in the 4-chamber view. The left atrial stiffness index (LASi) was obtained from the ratio between the E/E’ average and PALS. The following values were considered as reference ranges, according to the EACVI NORRE study [22,23]:

○ PALS: 42.5% (IQR 36.1–48.0%), LLN 26.1 ± 0.7%
○ LA conduit strain: −25.7% (IQR 20.4–31.8%), LLN—12.0 ± 0.5%
○ PACS: −16.3% (IQR 12.9–19.5%), LLN—7.7 ± 0.3%
○ LASi:
  - 0.12 (IQR 0.10–0.15) for ages 20–40, with LLN 0.22 ± 0.01
  - 0.13–0.22) for ages 40–60, with LLN 0.42 ± 0.04
  - 0.24 (0.18–0.29) for age > 60 years, with LLN 0.55 ± 0.09

### 2.5. Statistical Analysis

Continuous variables were expressed as means and standard deviations or medians with interquartile ranges as appropriate. The Student’s *t*-test or, when necessary, the Mann–Whitney test was used to compare the two groups. Categorical variables were evaluated based on percentages of the total population and compared using the χ^2^ test and Fisher’s exact test. A one-way ANOVA test was used to determine if there was a statistically significant difference between the means of three or more groups. After adjustment for lack of sphericity in repeated measures, the ANOVA was performed with the Greenhouse–Geisser correction. ROC analysis was performed to evaluate the predictive ability of the percentage reduction in LA strain value between T1 and T0 on the development of subclinical dysfunction, as assessed by a reduction in GLS > 15% from T0 to T1. 

A two-tailed *p*-value < 0.05 was considered statistically significant. Bonferroni’s correction was used to correct the significance of multiple tests. All the statistical analyses were performed using RStudio software (version 1.4.1103 2009–2021 RStudio).

## 3. Results

After collecting and analyzing data from the different centers involved, the “total study population” consisted of 169 patients (100% women, mean age: 55 ± 10.8 years) with newly diagnosed breast cancer, “HER2 receptor-positive”. According to the TNM classification, most patients were in stage IIA. More precisely, the anatomical classification according to TNM is shown in Table 1. As regards baseline cardiovascular risk factors, 22% (37 patients) had diabetes mellitus, 34% (57 patients) had arterial hypertension, 29% (50 patients) had obesity, and 33% (55 patients) were smokers. The hypertensive patients were treated with angiotensin-converting enzyme (ACE) inhibitors/angiotensin receptor blockers (ARBs), beta-blockers, or calcium channel blockers. Other basic clinical data of the study population are summarized in Table 1.

As for echocardiographic parameters at baseline, our cohort showed normal values of LVEF (60 ± 1.7%), GLS (−20.7 ± 2.1%), PALS (36 ± 8.9%), and normal values of LAVi and normal right ventricular function. Regarding the assessment of diastolic function in all the population at baseline, we found that at T0, 37% of patients had a normal diastolic function, 62% had grade I diastolic dysfunction, and only 1 patient had grade II diastolic dysfunction; no patients had grade 3 diastolic dysfunction. Table 2 summarizes key echocardiographic data from the total study population at baseline and during follow-up.

### 3.1. Follow-Up

**Figure 1:**
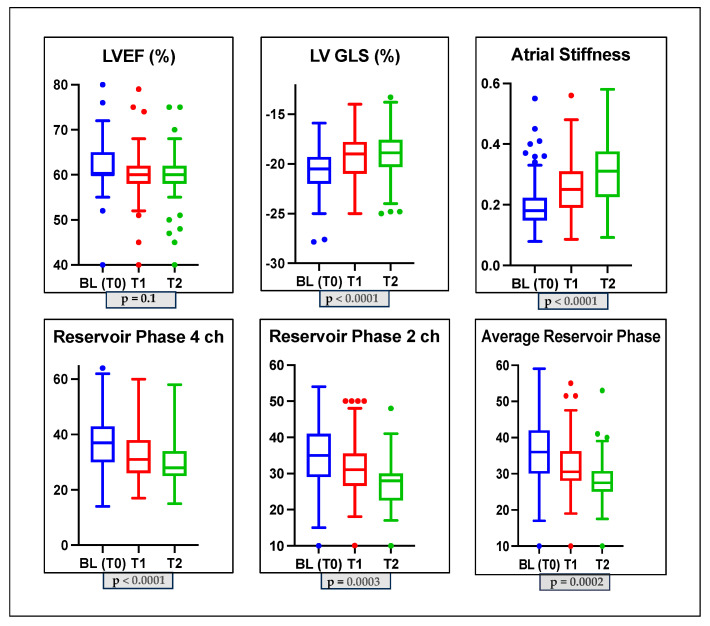
Box plots showing echocardiographic characteristics of the study population at baseline and during monitoring. LVGLS: left ventricle global longitudinal strain; LVEF: left ventricle ejection fraction; 2CH: two-chamber; 4CH: four-chamber.

In the whole population after 3 (T1) and 6 (T2) months of anti-cancer therapy, we did not observe a significant reduction in LVEF (60 ± 1.7 vs. 59.7 ± 2.8 vs. 59 ± 3.4; *p*-value > 0.05). Analyzing diastolic function, the percentages of diastolic dysfunction present at baseline (T0) remained unchanged at T1; at T2, only 1 patient had grade II diastolic dysfunction, 74% of the patients had grade I diastolic dysfunction, and 25% of the patients had no diastolic dysfunction. In addition, during the follow-up, the E/A ratio changed significantly (*p*-value = 0.01), but the mean E/E’ ratio (*p*-value = 0.3) and pulmonary systolic pressure did not change significantly (*p*-value = 0.4); see Table 2 and Figure 1. Conversely, all STE parameters changed significantly during the follow-up at T1 and T2. We observed a significant reduction in GLS (−20.7 ± 2.1 vs. −19.2 ± 2.5 vs. −18.9 ± 2.4 *p*-value < 0.0001) and in PALS (4-chamber LA reservoir strain: 37 ± 9.3 vs. 32.7 ± 9 vs. 29.6 ± 7.6, *p* ≤ 0.0001; 2-chamber LA reservoir strain: 36 ± 9.1 vs. 32 ± 7.7 vs. 27.8 ± 5.9, *p* = 0.0003; and average LA reservoir strain: 36 ± 8.9 vs. 32.4 ± 7.7 vs. 28.7 ± 6.3, *p* = 0.0002). Furthermore, LASi worsened during follow-up (0.19 ± 0.07 vs. 0.25 ± 0.08 vs. 0.30 ± 0.09; *p* < 0.0001); see Table 2. We also found a significant increase in LAVi during follow-up (*p*-value = 0.03). We evaluated the development of CTRCD during follow-up, and we found that no patients developed symptomatic CTRCD or severe asymptomatic CTRCD. One patient developed moderate asymptomatic CTRCD at T1, and two patients developed moderate asymptomatic CTRCD at T2. A total of 28 patients (17% of our total population) developed asymptomatic mild CTRCD.

### 3.2. Patients with and without Mild CTRCD

In Table 3 and Table 4, the main echocardiographic changes that occurred in patients with mild asymptomatic CTRCD (Group 1) and without CTRCD (Group 2), respectively, are summarized whose baseline characteristics are highlighted in Table 1.

**Figure 2:**
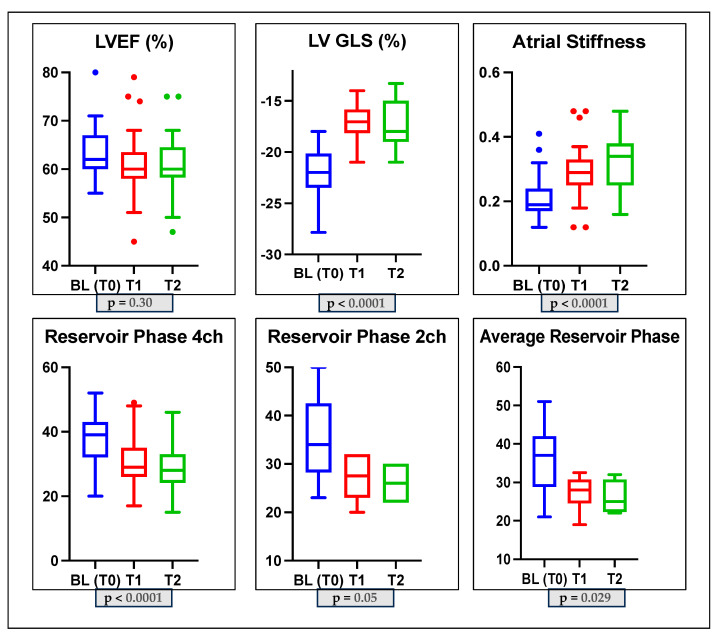
Box plots showing echocardiographic characteristics of the subclinical dysfunction group (Group 1) at baseline and during monitoring. LVGLS: left ventricle global longitudinal strain; LVEF: left ventricle ejection fraction; 2CH: two-chamber; 4CH: four-chamber.

Specifically, we found in Group 1 a non-significant reduction in LVEF during the follow-up (*p*-value = 0.30); a significant reduction in GLS over time (*p*-value < 0.0001); a significant reduction in PALS (4-ch: 37.5 ± 7.6 vs. 30.4 ± 8.3 vs. 29.3 ± 7.3 *p*-value < 0.0001; 2-ch: 35.5 ± 9.1 vs. 27.3 ± 4.8 vs. 26 ± 4.6, *p*-value = 0.05; average PALS: 35.9 ± 8.7 vs. 27.3 ± 4.4 vs. 25.6 ± 4.4 *p*-value = 0.029); and a significant increase in LASi (0.21 ± 0.06 vs. 0.29 ± 0.09 vs. 0.32 ± 0.08 *p*-value < 0.0001). The LAVi and mean E/e’ ratio did not change significantly (*p*-value > 0.05); the E/A ratio changed significantly (*p*-value = 0.04); see Table 3 and Figure 2.

Analyzing Group 2 (patients without asymptomatic mild CTRCD), we did not find significant changes in LVEF. We also observed significant changes in GLS (*p*-value = 0.0030), PALS (*p*-value < 0.0001), LASi (*p*-value < 0.0001), and mean E/e’ ratio (*p*-value = 0.01; see Table 4). However, it is worthy of notice that impairment of ventricular and atrial function was greater in Group 1 compared to Group 2 over time (Table 5).

In particular, we found a 20% reduction in PALS in Group 1 at T1 compared to baseline (Table 5). This reduction could be used as a cut-off to delimit patients who are more likely to develop subclinical cardiac dysfunction, as assessed by a relative reduction in GLS > 15% while taking anticancer drugs. In fact, we found in the ROC curve analysis that PALS variation > 20.8% was fairly accurate in identifying patients who were most likely to develop asymptomatic mild cardiotoxicity [AUC 0.62; CI (0.51–0.73) *p* = 0.06, sensitivity 45%, specificity 69.5%].

In addition, when comparing the two groups at baseline, we found no significant differences in baseline PALS value or other echocardiographic parameters; only the baseline GLS and mean E/e’ ratio differed significantly but remained within normal values (Table 6).

## 4. Discussion

The present multicenter study was one of the first to evaluate LA function by STE in cardio-oncology. Our study showed the cardiotoxic effects of anthracyclines and trastuzumab on atrial function evaluated by STE, and it identified a percent change in PALS able to detect patients at higher risk of CTRCD. In our study, GLS and PALS changed significantly in all the patients treated with anti-cancer drugs, LASi increased significantly, and LVEF did not change during follow-up. Thus, in agreement with the literature data, our study reconfirms the ability of deformation imaging parameters such as GLS to identify signs of cardiotoxicity. In fact, GLS is more sensitive than LVEF and other echocardiographic parameters such as TDI for detecting early cardiac damage [24]. For a long time, LVEF has been used as the only tool for diagnosing cardiotoxicity. Beyond LVEF, research has moved towards finding new parameters that could help predict subclinical dysfunction. Among the methods developed in the search for subclinical cardiotoxicity, STE is certainly one of the most interesting and promising, because it allows a post-processing evaluation through the use of dedicated software [25]. Furthermore, the low costs, the reduced intra- and inter-operator variability (inter-examination variability of about 6% for GLS versus 10% for LVEF), together with the standardization of cut-off values and measurement methods promoted by the main scientific societies (EACVI, ASE, ESC) and by the manufacturers have allowed the considerable development of this method and its wide diffusion in echocardiography laboratories [26]. Myocardial work and other echocardiographic parameters are also emerging as early markers of cardiotoxicity [27,28,29]. Few studies have evaluated LA strain as a marker of early cardiac dysfunction without clear results on the usefulness of this parameter in predicting cardiotoxicity [30]. A study in the adult cancer population found no significant changes in LA strain during anthracycline chemotherapy [31]. On the other hand, another study found a significant and frequent reduction in LA strain after doxorubicin therapy and even suggested LA strain as being a more sensitive marker than LVEF or GLS in detecting CTRCD [32,33]. In particular, Laufer-Perl et al. showed that reduction in LA reservoir strain and LA conduit strain is frequent and occurs early in the course of anthracycline therapy, showing a significant correlation with routine echocardiographic diastolic parameters [32]. Other studies in cardio-oncology have shown significant changes in diastolic function; abnormal or worsening diastolic function precedes systolic dysfunction, and it is associated with an increased risk of CTRCD in patients with breast cancer [34]. GLS and LA strain are closely related parameters. The evaluation of left ventricular function cannot be separated from the evaluation of atrial function. Indeed, the LA function modulates LV filling and plays an important role in the diastolic phase of the cardiac cycle, while the LV function affects LA contraction and relaxation. Thus, changes in the intracavitary pressures in one chamber also affect the other [35]. In our study, we observed in all the patients a reduction in GLS and PALS and an increase in LASi in the presence of normal LVEF and normal left ventricular filling pressures. The changes in PALS and LASi were greater in the patients with CTRCD. A reduction in PALS > 20.8% (assessed only in the 4-chamber view) has been identified as a cut-off to detect patients at higher risk of CTRCD, with the best sensitivity and specificity. Decreased GLS and worsening LVEF are known to be associated with increased left ventricular filling pressures and decreased PALS. PALS has many advantages: it is angle-independent, overcoming Doppler limitations, and it provides a reproducible measurement of the LA deformation. It also helps to define indeterminate cases of diastolic dysfunctions [36]. In addition, the LA function can be considered a reliable index to estimate the diastolic function of the left ventricle [37]. Moreover, it is a strong predictor of functional capacity and an important prognostic marker in various diseases such as atrial fibrillation, mitral regurgitation, and HFpEF [38,39,40]. We believe that cardiotoxicity induced by antineoplastic drugs is a comprehensive phenomenon involving, contemporaneously, the whole heart (either ventricle or atria); accordingly, we detected by speckle-tracking analysis an impairment of either atrial or ventricular function. According to our results, LA function assessed by STE could be used, not as a substitute but as an adjunctive parameter to GLS in the assessment of patients undergoing treatment with anti-cancer drugs to identify early signs of cardiotoxicity. The main limitation of our study was the short follow-up time, which did not allow us to establish the real prognostic power of the detected left atrial function abnormalities in terms of LVEF decline and the occurrence of adverse events. A second limitation of the study was the low-risk profile of the study population, which, however, was an inclusion criterion. Another limitation of our study was the lack of determination of biomarkers to assess cardiotoxicity, due to the variability of the methods used for determination in the various centers. In addition, other limitations were the use of non-dedicated software for the analysis of LA strain and the use of the same software for the left ventricle. Moreover, for the PALS assessment, we employed QRS, and not P wave, as the reference point given that it represents the most used method.

## 5. Conclusions

Our multicenter study highlights the usefulness of LA function assessment using STE in women with breast cancer treated with chemotherapy because atrial function also changes significantly during anticancer treatment. Particularly in our study, the impairment of LA strain was significantly higher in patients who developed asymptomatic mild CTRCD. A cut-off of reduction in PALS of 20.8% was identified to detect patients at higher risk of cardiotoxicity. Thus, the assessment of PALS in addition to GLS could be considered to discover subclinical cardiotoxicity more accurately. In daily clinical practice, the use of this parameter could help in identifying cancer patients with a greater risk of cardiotoxicity to more precisely manage cardioprotective therapy and design tailored cardiological follow-ups.

## Figures and Tables

**Table 1:** General characteristics of the study population and subgroups.

MEAN ± SD	Overall Population n = 169	Group 1 n = 28	Group 2 n = 141
Age (years)	55 ± 10.8	55 ± 10.7	53.3 ± 9.7
Weight (Kg)	65.8 ± 11.8	62 ± 10.8	66.7 ± 11.8
Body Surface Area BSA (m^2^)	1.7 ± 0.14	1.6 ± 0.12	1.7 ± 0.15
Body Mass Index BMI (Kg/m^2^)	25.1 ± 4.5	24.8 ± 4.3	25 ± 4.4
Diabetics % (n)	22% (37)	14% (4)	18.4% (26)
Hypertensive % (n)	34% (57)	25% (7)	26.2% (37)
Obesity % (n)	29% (50)	11% (3)	25.5% (36)
Family history of cardiovascular disease % (n)	26% (44)	14% (4)	21.2% (30)
Smokers % (n)	33% (55)	50% (14)	55.3% (78)
Ex-smokers % (n)	10% (17)	14% (4)	5.7% (8)
T1N1M0	45.5% (77)	21.4% (6)	50.3% (71)
T2N0M0	15.4% (26)	25% (7)	13.5% (19)
T2N1M0	39% (66)	53.5% (15)	36.1% (51)

**Table 2:** Echocardiographic characteristics of the study population at baseline and during monitoring.

n = 169	MEAN (SD)	T0 (BL)	T1	T2	*p*-Value
LEFT VENTRICLE	EDV (ML)	80.6 (16)	84.9 (13.6)	84.1 (14.8)	0.7
	EF (%)	60 (1.7)	59.7 (2.8)	59 (3.4)	0.1
	GLS (%)	−20.7 (2.1)	−19.2 (2.5)	−18.9 (2.4)	<0.0001
LEFT ATRIAL	VOLUME (ML)	46 (14)	47 (12.8)	46 (13.8)	0.1
	VOLUME INDEXED (ML/MQ)	27.9 (7.3)	27.7 (9.1)	30.3 (7.3)	0.03
LEFT ATRIAL FUNCTION	RESERVOIR PHASE 4CH	37 (9.3)	32.7 (9)	29.6 (7.6)	<0.0001
	RESERVOIR PHASE 2CH	36 (9.1)	32 (7.7)	27.8 (5.9)	0.0003
	AVERAGE RESERVOIR PHASE	36 (8.9)	32.4 (7.7)	28.7 (6.3)	0.0002
	ATRIAL STIFFNESS	0.19 (0.07)	0.25 (0.08)	0.30 (0.09)	<0.0001
TISSUE DOPPLER IMAGING (TDI)	E WAVE (CM/SEC)	67.7 (24.2)	75.7 (16.7)	74.7 (16.9)	0.3
	E/A	1 (0.3)	0.97 (0.2)	1.1 (0.3)	0.01
	E/E’	7.2 (2.3)	7.5 (1.9)	8 (2.7)	0.3
PULMONARY PRESSURE	SPAP (MMHG)	26 (4.5)	23.2 (4.7)	23.5 (4)	0.4
RIGHT VENTRICLE FUNCTION	TAPSE (MM)	22.1 (3)	21.6 (3.3)	21.7 (2.5)	0.1
	S’ TDI (CM/SEC)	13 (2)	13 (2.3)	12.6 (1.6)	0.2

EDV: end-diastolic volume; EF: ejection fraction; GLS: global longitudinal strain; SPAP: systolic pulmonary artery pressure; TAPSE: tricuspid annular plane systolic excursion; TDI: tissue doppler imaging; 2CH: two-chamber; 4CH: four-chamber.

**Table 3:** Echocardiographic characteristics in the subgroup of patients with asymptomatic mild CTRCD (Group 1).

GROUP 1 (n = 28)	MEAN (SD)	T0 (BL)	T1	T2	*p*-Value
LEFT VENTRICLE	EDV (ML)	76.4 (10.7)	82.4 (15.3)	77.3 (12.7)	0.08
	EF (%)	60.1 (1.5)	59.2 (4)	59.4 (4)	0.30
	GLS (%)	−21.9 (2.1)	−17 (1.6)	−17.3 (2.2)	<0.0001
LEFT ATRIAL	VOLUME (ML)	47.2 (12.4)	52.2 (10.6)	50.5 (10.2)	0.3
	VOLUME INDEXED (ML/MQ)	28 (7.7)	31.4 (7)	31.4 (6)	0.26
LEFT ATRIAL FUNCTION	RESERVOIR PHASE 4CH	37.5 (7.6)	30.4 (8.3)	29.3 (7.3)	0.0001
	RESERVOIR PHASE 2CH	35.5 (9.1)	27.3 (4.8)	26 (4.6)	0.05
	AVERAGE RESERVOIR PHASE	35.9 (8.7)	27.3 (4.4)	25.6 (4.4)	0.029
	ATRIAL STIFFNESS	0.21 (0.06)	0.29 (0.09)	0.32 (0.08)	<0.0001
TISSUE DOPPLER IMAGING (TDI)	E WAVE (CM/SEC)	83.5 (20.6)	76.8 (20.8)	75 (20.7)	0.22
	E/A	1.1 (0.33)	0.97 (0.25)	1.1 (0.32)	0.04
	E/E’	8 (1.7)	8.52 (2.7)	8.9 (3)	0.1
PULMONARY PRESSURE	SPAP (MMHG)	24.6 (5.8)	26.8 (4.9)	27.4 (5.7)	0.5
RIGHT VENTRICLE FUNCTION	TAPSE (MM)	22.6 (3)	20.8 (4.3)	21.2 (2.9)	0.1
	S’ TDI (CM/SEC)	12 (1.5)	12.4 (2.6)	13 (0.75)	0.5

EDV: end-diastolic volume; EF: left ventricle ejection fraction; GLS: global longitudinal strain; SPAP: systolic pulmonary artery pressure; TAPSE: tricuspid annular plane systolic excursion; TDI: tissue doppler imaging; 2CH: two-chamber; 4CH: four-chamber.

**Table 4:** Echocardiographic characteristics in the subgroup of patients without asymptomatic mild CTRCD (Group 2).

GROUP 2 (n = 141)	MEAN (SD)	T0 (BL)	T1	T2	*p*-Value
LEFT VENTRICLE	EDV (ML)	82.4 (18.2)	85 (13)	85.7 (15.4)	0.5
	EF (%)	60 (1.8)	60 (2.3)	59 (3.2)	0.05
	GLS (%)	−20.4 (2)	−20 (2.4)	−19.6 (2.3)	0.0030
LEFT ATRIAL	VOLUME (ML)	43.5 (14.3)	44.5 (13.4)	43.6 (12.3)	0.1
	VOLUME INDEXED (ML/MQ)	27.2 (7.7)	26.4 (10.2)	29.5 (6.3)	0.2
LEFT ATRIAL FUNCTION	RESERVOIR PHASE 4CH	37.3 (9.4)	33.8 (9.7)	29.9 (7.8)	<0.0001
	RESERVOIR PHASE 2CH	35.9 (9)	32.5 (7.7)	27.6 (6.1)	<0.0001
	AVERAGE RESERVOIR PHASE	35.6 (8.9)	33 (7.7)	28.8 (6.5)	<0.0001
	ATRIAL STIFFNESS	0.2 (0.08)	0.24 (0.08)	0.29 (0.09)	<0.0001
TISSUE DOPPLER IMAGING (TDI)	E WAVE (CM/SEC)	74.5 (16.1)	75.7 (16.1)	76.5 (15.7)	0.3
	E/A	1.1 (0.3)	0.97 (0.27)	1.1 (0.35)	0.08
	E/E’	7.2 (2.6)	7.3 (1.8)	8 (2.8)	0.01
PULMONARY PRESSURE	SPAP (MMHG)	23.7 (5.8)	22.4 (4.7)	22.7 (2.9)	0.2
RIGHT VENTRICLE FUNCTION	TAPSE (MM)	22 (3)	21.6 (3)	21.6 (2.4)	0.5
	S’ TDI (CM/SEC)	12.9 (1.9)	13 (2.3)	12.4 (1.5)	0.5

EDV: end-diastolic volume; EF: left ventricle ejection fraction; GLS: global longitudinal strain; SPAP: systolic pulmonary artery pressure; TAPSE: tricuspid annular plane systolic excursion; TDI: tissue doppler imaging; 2CH: two-chamber; 4CH: four-chamber.

**Table 5:** LVEF, GLS, atrial strain, and atrial stiffness changes in Group 1 compared to Group 2.

	SUBCLINICAL DYSFUNCTION GROUP GROUP 1	NO SUBCLINICAL DYSFUNCTION GROUP GROUP 2	*p*-Value
VARIATION OF LVEF BETWEEN T0 AND T1	−0.7 ± 2.9	−0.2 ± 2.7	0.38
VARIATION OF LVGLS BETWEEN T0 AND T1	+4.89 ± 1.5	+0.20 ± 2	<0.0001
RESERVOIR PHASE 4CH. T0	37.4 ± 7.58	37.2 ± 9.43	0.4
VARIATION OF PALS (4CH). BETWEEN T0 AND T1	−6.86 ± 10.1	−2.97 ± 6.9	0.02
% OF REDUCTION IN PALS (4CH) BETWEEN T0 AND T1	−20%	−8%	0.03
VARIATION OF PALS (2CH). BETWEEN T0 AND T1	−11 ± 8.7	−3.32 ± 4.5	0.0008
VARIATION OF PALS (AVERAGE) BETWEEN T0 AND T1	−8.3 ± 3.3	−4.7 ± 2.1	0.01
VARIATION OF ATRIAL STIFFNESS	+0.11 ± 0.05	+0.05 ± 0.08	0.0026

GLS: global longitudinal strain; LV: left ventricle; LVEF: left ventricle ejection fraction; PALS: peak atrial longitudinal strain; 2CH: two-chamber; 4CH: four-chamber.

**Table 6:** Echocardiographic characteristics at baseline in the 2 subgroups.

T0 (BL)	VARIABLES	GROUP 1 n = 28 MEAN (SD)	GROUP 2 n = 141 MEAN (SD)	*p*-Value
LEFT VENTRICLE	EDV (ML)	76.4 (10.7)	82.4 (18.2)	0.4
	EF (%)	63.3 (5.3)	62 (4.6)	0.2
	GLS (%)	−21.9 (2.1)	−20.4 (2)	0.0038
LEFT ATRIAL	VOLUME (ML)	47.2 (12.4)	43.5 (14.3)	0.5
	VOLUME INDEXED (ML/MQ)	28 (7.7)	27.2 (7.7)	0.9
LEFT ATRIAL FUNCTION	RESERVOIR PHASE 4CH	37.5 (7.6)	37.3 (9.4)	0.7
	RESERVOIR PHASE 2CH	35.5 (9.1)	35.9 (9)	0.8
	AVERAGE RESERVOIR PHASE	35.9 (8.7)	35.6 (8.9)	0.9
	ATRIAL STIFFNESS	0.21 (0.06)	0.2 (0.08)	0.3
TISSUE DOPPLER IMAGING (TDI)	E WAVE (CM/SEC)	83.5 (20.6)	74.5 (16.1)	0.3
	E/A	1.1 (0.33)	1.1 (0.3)	0.8
	E/E’	8 (1.7)	7.2 (2.6)	0.0045
PULMONARY PRESSURE	SPAP (MMHG)	24.6 (5.8)	23.7 (5.8)	0.9
RIGHT VENTRICLE FUNCTION	TAPSE (MM)	22.6 (3)	22 (3)	0.4
	S’ TDI (CM/SEC)	12 (1.5)	12.9 (1.9)	0.1

EDV: end-diastolic volume; GLS: global longitudinal strain; LVEF: left ventricle ejection fraction; SPAP: systolic pulmonary artery pressure; TAPSE: tricuspid annular plane systolic excursion; TDI: tissue doppler imaging; 2CH: two-chamber; 4CH: four-chamber.

## Data Availability

Data will be available upon reasonable request.
